# Regional versus local wind speed and direction at a narrow beach with a high and steep foredune

**DOI:** 10.1371/journal.pone.0226983

**Published:** 2020-01-02

**Authors:** Winnie de Winter, Jasper Donker, Geert Sterk, Job van Beem, Gerben Ruessink

**Affiliations:** Department of Physical Geography, Faculty of Geosciences, Utrecht University, Utrecht, The Netherlands; Universidade de Aveiro, PORTUGAL

## Abstract

Dune growth and post-storm recovery of foredune systems is predominantly determined by the aeolian sand transport through the beach-dune interface. Potential sand transport rates, estimated with empirical transport equations using regionally representative wind conditions, are generally too high. This positive bias might be, at least partly, due to the effect of the beach and foredune topography on the regional airflow. Here, we investigate the relation between local (on the beach) and regional wind velocities and direction in front of the high (∼22 m) and steep (∼1:2.5) foredune partially vegetated with Marram grass at Egmond aan Zee, The Netherlands based on a dataset with a large variety in wind speeds spanning over all onshore wind directions. We observed that local 10-minute averaged wind speed and direction can differ from the regional wind conditions (here measured 15 km away from the study site) depending on the regional approach angle of the wind. The ratio of local over regional wind speed is smallest (∼0.39) when the wind direction is dune-normal. This ratio increases with increasing obliquity towards almost 1 for alongshore winds. Wind steering only happens at the dune foot and is the largest (∼13°) with oblique approaching winds of 40° from the dune normal. Perpendicular and nearly alongshore winds do not show any steering near the dune foot. The use of local rather than regional wind conditions in a potential transport equation reduces the predicted annual supply from 86 to 32 *m*^3^/*m*/*y*, substantially closer to the measured deposition of 15 *m*^3^/*m*/*y*. The drop in velocity was more important to the reduction in predicted supply than the alongshore steering.

## Introduction

Foredunes protect a large portion of the worlds coastal areas from marine flooding during severe storms. Additional to safety, foredunes contain important habitats for coastal flora and fauna and the dynamics of foredunes can sustain high biodiversity in the entire dune system. Duneward aeolian transport from the beach is essential for the growth, dynamics and post-storm recovery of foredune systems [1, 2]. Commonly used aeolian sand transport models compute the sand transport rate with the shear velocity to the third power [3–10], where the shear velocity is spatially constant and based on time-averaged wind velocity measurements. Predictions of sand transport through the beach-dune interface are generally too high. The issue of the alteration of regional flow velocity and direction due to local topography and the consequences for the local aeolian sediment transport rate has been observed before [11]. [2, 12, 13] found that the magnitude of the potential sand transport rates exceed the actual transport rates measured in the field in the order of 25 to 50% [12, 14, 15]. For example, predictions of potential transport for high (larger than 20 m) and steep (smaller than 1:2.5) coastal foredune systems in the Netherlands are larger than 50 *m*^3^/*m*/*y* and the mean annual transport potential is 125 *m*^3^/*m* [2], whereas the actual deposition is much lower and in the order of 15 *m*^3^/*m*/*y* [13, 16].

The mismatch is commonly ascribed to supply limiting conditions (e.g. grain size, soil moisture content, surface roughness, the amount of shell fragments and fetch length) [17–23] and weather conditions such as rainfall [24]. In very few instances the mismatch has been linked to topographic acceleration or deceleration of the wind [11]. However, this disregards the modification of wind flow due to the topography of the beach and foredune [25–27] when wind conditions measured at a regional weather station, often 10 km-s or more away from the actual foredune system under study, are used in aeolian transport rate models. Wind speed over escarpments has been investigated in windtunnel studies [28–30], with model studies [31–33] and in field studies [27, 29, 34–37]. Large non-aerodynamic objects cause a build up of pressure on their upwind side which leads to a smooth transition of streamlines around the object and reduced wind velocities in the high pressure area [29]. This means that at the dune foot and beach local wind speeds should be lower compared to a regionally representative wind speed due to the built up of pressure in front of the foredune. This effect is expected to increase with higher and steeper foredunes [27, 38, 39], because this would create a larger pressure build up. In addition, the reduction of local wind speed is dependent on the approach angle [27, 39], since the effective slope decreases with increasing obliquity [26]. The studies mentioned above mainly focused on the down-wind side of a foredune, while studying the upwind side of the foredune (beach and dune foot) would contribute to knowledge on wind forcing in the area where sand transport is initiated. Additionally, the abundance of field data is missing in these model and conceptual studies. Due to the topography-related decrease in local wind speed, the use of regional wind speed can contribute to positive bias of the potential with respect to actual sand transport rate.

The magnitude of potential onshore sand transport can be approached using the ‘cosine effect’ [12] at the beach-dune interface. By using this concept the onshore component of the sand transport vector is calculated based on regional wind directions, using cos(*θ*_*reg*_) where *θ*_*reg*_ is the regional wind angle with respect to the dune normal. The wind direction may be spatially variable due to observations on topographic steering or deflection of the wind over a foredune [25, 40]. Conceptual plots of [27] and [39] show that deflection also occurs at the dune foot and steers the wind in a more alongshore direction. This is confirmed by the orientation of observed aeolian bed forms near the dune foot [41]. However, few observations herein exist specifically on the beach and at the dune foot and on the magnitude of steering. When the wind is steered alongshore at the beach-dune interface, it would increase the obliquity of the wind and thus decrease the onshore component of the sand transport vector. Ignoring alongshore steering would thus lead to an overestimation of the potential deposition at the foredune.

The aim of this paper is to investigate wind speed and direction upwind of a steep (22°) and tall (∼22 m above mean sea level) foredune at Egmond aan Zee, The Netherlands. The main focus is on the relation of these wind characteristics at the beach to regionally measured wind data from a closeby weather station and is elaborated with the spatial variability at the beach itself. The data were collected during two 6-week field campaigns of wind measurements comprising a high variety in (regional) speeds and directions. We elaborate on the effect of the use of regional versus local wind conditions on the prediction of the potential aeolian transport rate for the present study site. The discussion and conclusions are provided in the final two sections of this paper.

## Methods

### Site description

**Fig 1:**
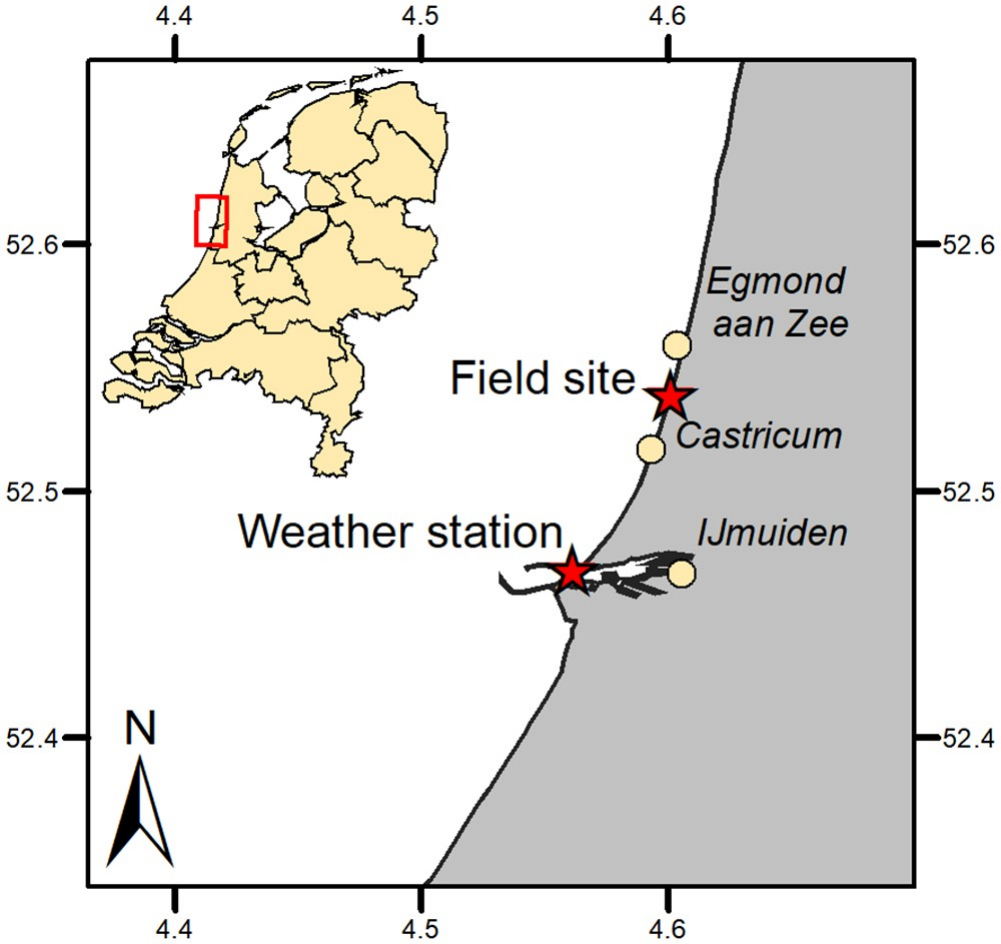
Locations of data collection at the field site near Egmond aan Zee and the KNMI weather station at IJmuiden. The red stars indicate the location of the field site (local wind measurements) and the KNMI weather station at IJmuiden (regional wind measurements). Reprinted from Esri Netherlands & Community Maps providers under a CC BY license, with permission from Esri Netherlands, original copyright 2019.

**Fig 2:**
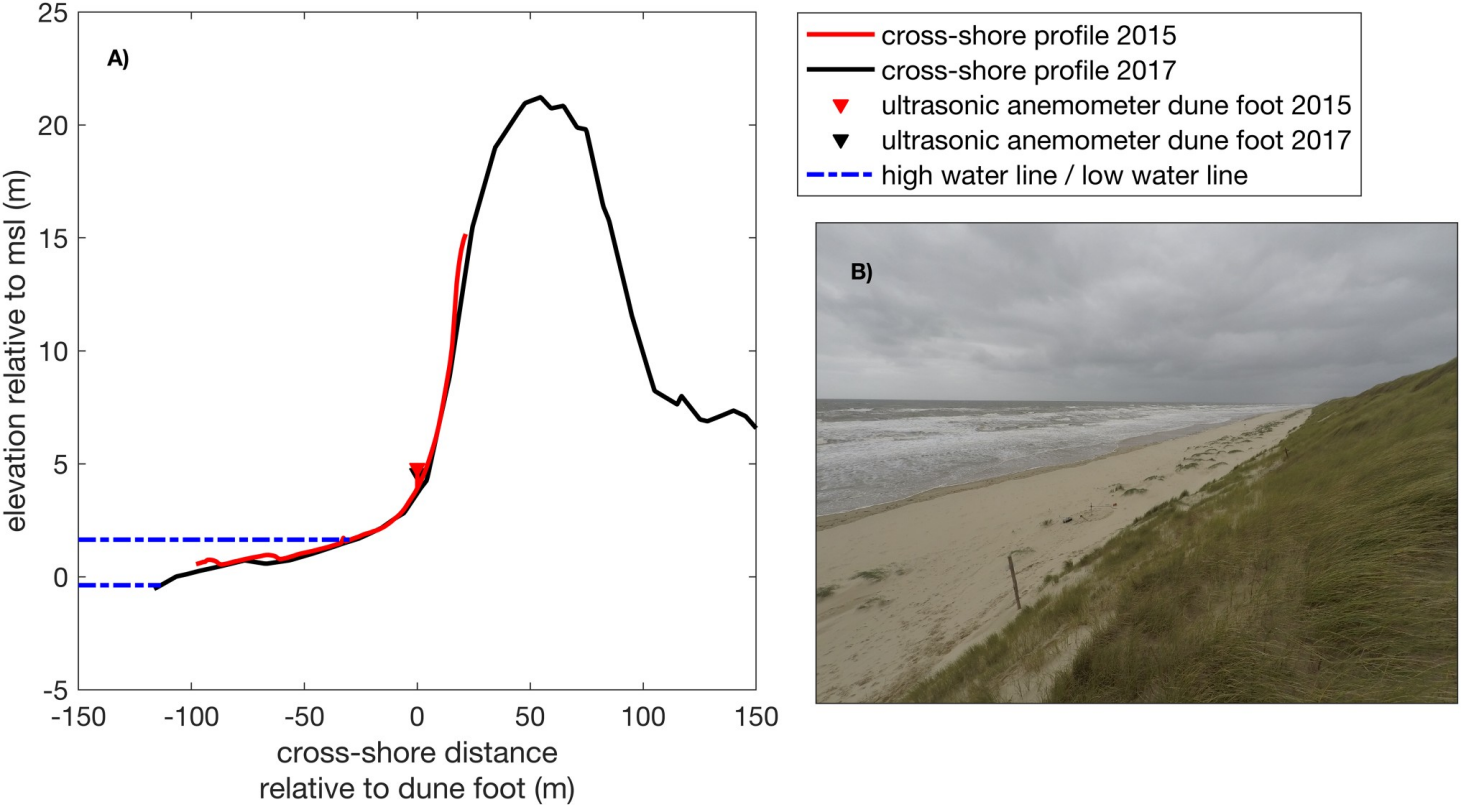
Morphological conditions at the beach near Egmond aan Zee. A: cross-shore profile of the field site in 2015 (red) and 2017 (black) and B: photo of the beach with the vegetated embryonic dunes (0.8—1.0 m height), south of the cross-shore measurement array of the field campaign in 2017.

All field experiments were conducted approximately 3 kilometers south of the beach town Egmond aan Zee, The Netherlands (Fig 1). During the study period, the morphology comprises an intertidal bar and trough on a moderately sloping (1:30) accretive beach. Downwind of our area of interest (beach) a steep foredune front (1:2.5 or 22°) is present that extends to approximately 22 m above mean sea level (Fig 2A) and is partially covered with Marram grass (*Ammophila arenaria* L.). The steep foredune front resulted from earlier erosion events [42]. Dune erosion typically occurs during strong northwesterly winds leading to storm waves larger than 5 *m* and surges larger than 1 *m* due to the large fetch over the North Sea basin from this direction. Embryo dunes can develop at the beach-dune interface during prolonged periods without erosion. The median grain size (D50) of the quartz sands on the beach is ∼250 *μ* *m*. Depending on the tide (microtidal range is between 1.2 and 1.8 *m*), the beach width varies between 30 and 100 m. The orientation of this relatively narrow beach deviates 7.2° east from the north and the dune front parallel to the coast is more or less straight.

**Fig 3:**
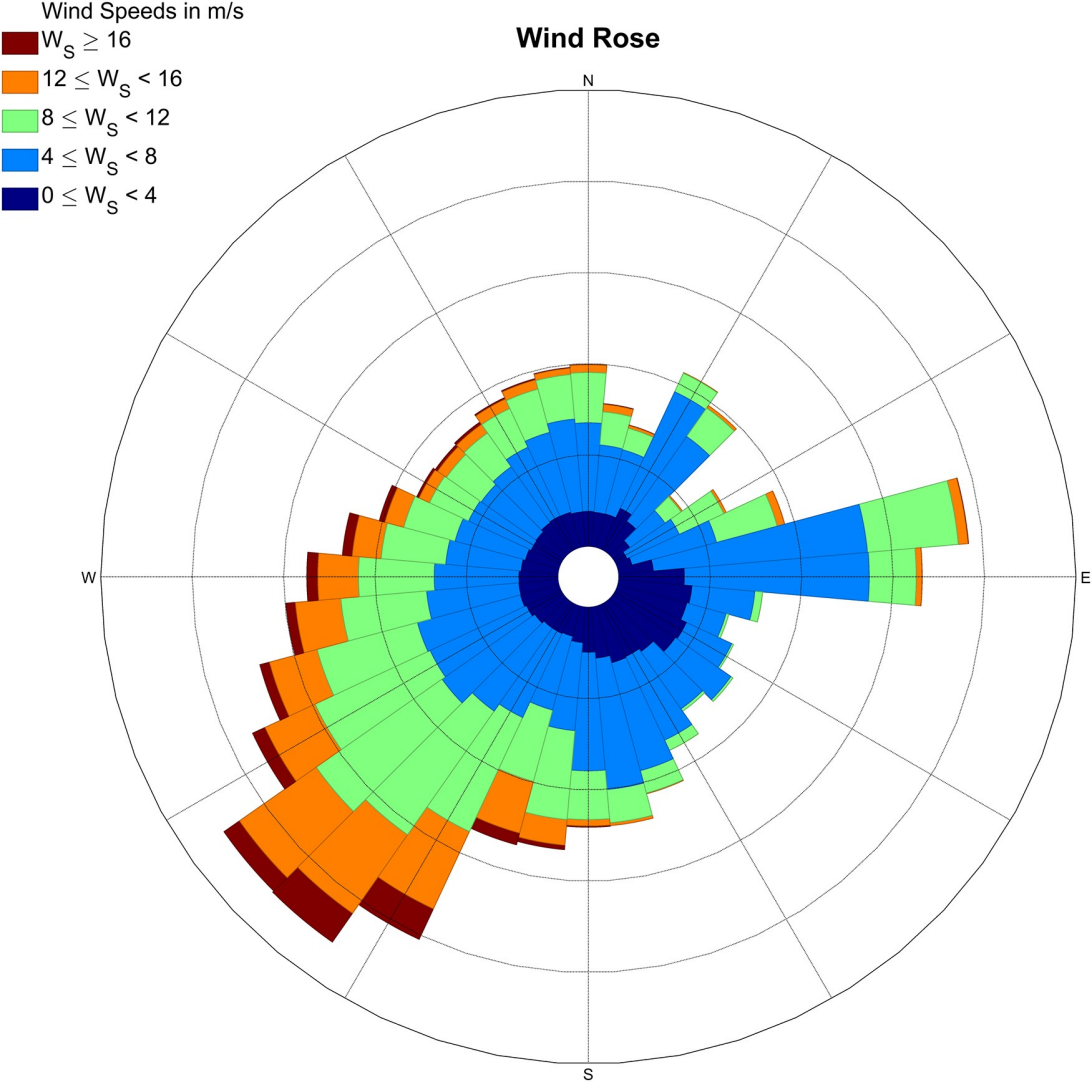
Wind rose at the IJmuiden weather station based on 10-minute data from January 1, 2007 up to and including December 31, 2016.

Regional wind conditions are measured at a meteorological station of the Dutch national weather service KNMI (Royal Netherlands Meteorological Institute) in IJmuiden (World Meteorological Organization (WMO) 06225, N 52° 27.733’, E 004° 33.300’), The Netherlands (Fig 1). This weather station is located approximately 15 km south of our field site at the end of the southern IJmuiden harbour mole, close to the local beach-foredune transition. The data in Fig 3 were based on 10-minute average values at a height of 10 m above ground level collected in 2007-2017. In this time period the annual mean wind speed was between 3.7 and 5.8 *m*/*s* and the maximum 10-minute mean wind speed was 30 *m*/*s*. The largest wind speeds of the 5 percentile exceedence in this 10-year time period are larger than 15 *m*/*s*. The modal wind direction is 225-230° from North and thus the wind climate is dominated by oblique-onshore (south-west to south-south-westerly) winds (Fig 3).

### Field experiment

**Fig 4:**
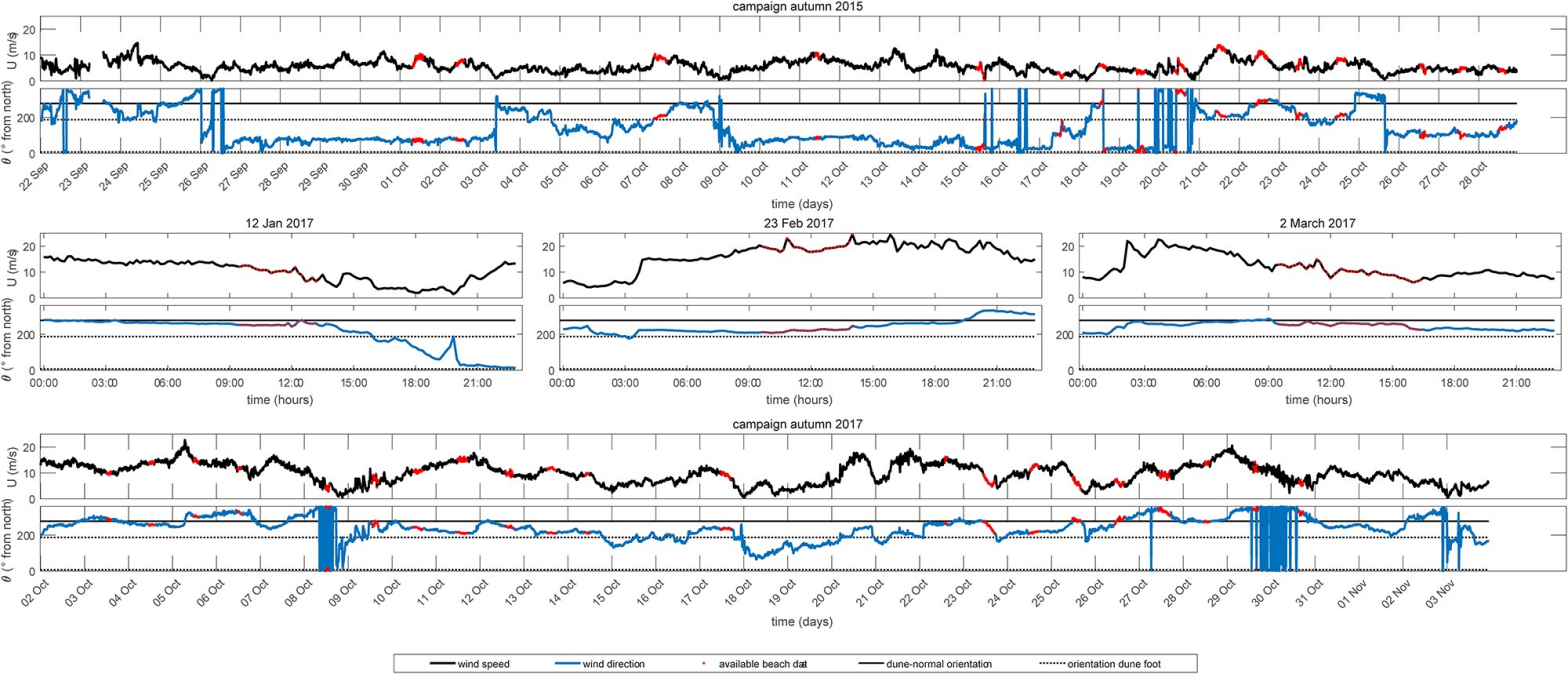
Wind conditions (speed and direction) measured at IJmuiden during all field experiments. Wind speed is plotted in black and wind direction in blue. The cross-shore and alongshore orientations are indicated with a black-continuous and a black-dotted line, respectively. The red dots indicate the moments when the cross-shore array of ultrasonic anemometers was deployed.

To carry out our field experiment we obtained permission at The Directorate-General for Public Works and Water Management (Dutch: Rijkswaterstaat). Field experiments were conducted during two 6-week field campaigns in autumn 2015 and 2017 (September 22 to October 30 and October 2 to November 3, respectively). Wind measurements were carried out, primarily during daytime (Fig 4). Additionally, wind measurements were taken on 3 separate days during the winter of 2017 (January 12, February 23 and March 2) because of severe storm conditions. During these measurement campaigns we measured regional wind speeds between 0 and 18 *m*/*s* and regional wind directions over the full onshore range. Depending on the tidal water level, and thus beach width, between 3 and 6 ultrasonic anemometers were deployed in a cross-shore array from the waterline to the dune foot with a spacing of about 10 m. The sampling frequency was 10 Hz and the measuring height was 0.9 m to prevent damage of the ultrasonic anemometer due to grains in saltation. One of the ultrasonic anemometers had a fixed location at the dune foot (≈ 3 m above mean sea level) and is used here as a local reference station. Approaching low tide, the cross-shore array was often extended with one additional ultrasonic anemometer on the upper dry beach, one at the high water line and two to three ultrasonic anemometers in the intertidal area on top of the intertidal bar and/or inside the through. All ultrasonic anemometers were aligned to a reference beach pole (with known coordinates), using a look-through-scope against the 0° and 120° sensor radii of the ultrasonic anemometer. Additionally, the ultrasonic anemometers were leveled using a level on top of the instrument. Cross-shore morphological surface profiles were measured relative to mean sea level using a RTK GPS system attached to a survey wheel. We used measurements from different field campaigns in a timeframe of 2 years, consequently a difference in the development stage of the embryonic dunes was observed. In autumn 2015 small (∼ 0.5 m), mainly non-vegetated, embryonic dunes were present, whereas in the fall of 2017 vegetated and larger (0.8–1 m height) embryonic dunes were present mainly south of our measurement array (Fig 2B).

### Data processing and analysis

Locally observed velocity data (*u*_*x*_, *v*_*y*_ and *w*_*z*_, respectively stream wise, perpendicular to stream wise and vertical velocities), measured in the internal coordinate system of the ultrasonic anemometer, were rotated for yaw, pitch and roll to align the velocities to the local streamlines [43]. Yaw rotation included the rotation in the horizontal *x*-*y*-plane to align the *u* velocity toward the mean flow vector using the following equations:

$$\begin{array}{c} \theta =\tan^{-1}(\frac{\bar{v_{y}}}{\bar{u_{x}}}) \end{array}$$ (1)

$$\begin{array}{c} u_{1}=u_{x}\cos\theta +v_{y}\sin\theta \end{array}$$ (2)

$$\begin{array}{c} v_{1}=-u_{x}\sin\theta +v_{y}\cos\theta \end{array}$$ (3)

where *θ* is the time-averaged flow angle, *u*_1_ and *v*_1_ are the instantaneous yaw-corrected velocity values (*m*/*s*), and the overlines indicate 10-minute averaged values. Pitch rotation was then applied to orient the *u* velocity parallel to the streamlines using

$$\begin{array}{c} \phi =\tan^{-1}(\frac{\bar{w_{z}}}{\bar{u_{1}}}) \end{array}$$ (4)

$$\begin{array}{c} u_{2}=u_{1}\cos\phi +w_{z}\sin\phi \end{array}$$ (5)

$$\begin{array}{c} w_{2}=-u_{1}\sin\phi +w_{z}\cos\phi \end{array}$$ (6)

where *ϕ* is the angle between the horizontal plane and the streamline, and *u*_2_ and *v*_2_ are the instantaneous yaw-pitch-corrected values (*m*/*s*). Because the beach had a sloping surface and the ultrasonic anemometers were level-installed, we used roll rotation to orient *v* along and *w* perpendicular to the stream surfaces as

$$\begin{array}{c} \psi =\frac{1}{2}\tan^{-1}(\frac{2\bar{v_{1}w_{2}}}{\bar{v_{1}^{2}}-\bar{w_{2}^{2}}}) \end{array}$$ (7)

$$\begin{array}{c} v_{3}=v_{1}\cos\psi +w_{2}\sin\psi \end{array}$$ (8)

$$\begin{array}{c} w_{3}=-v_{1}\sin\psi +w_{2}\cos\psi \end{array}$$ (9)

where *ψ* is the roll rotation angle, and *v*_3_ and *w*_3_ are the instantaneous yaw-pitch-roll-corrected values (*m*/*s*). The resulting three dimensional velocities *u*_2_, *v*_3_ and *w*_3_ were used for further analysis and are referred to as *u*, *v* and *w*, respectively from now on. The 10-minute average wind speed is $\bar{u}$ (streamwise velocity), as $\bar{v}$ (perpendicular velocity) and $\bar{w}$ (vertical velocity) are 0 by definition.

The wind direction in each 10-minute data block with respect to north were derived using Eq (1) and corrected for the alignment of the ultrasonic anemometer in the field, where the alignment relative to a beach pole (with known coordinates) was determined using the Pythagorean theorem and was corrected with 30° for the scope alignment relative to the internal axes of the ultrasonic anemometer.

To compare the locally measured wind data with the regional wind data of the IJmuiden weather station we transformed the 10-minute average wind velocities, measured at 10 m to a height of 0.9 m, using a logarithmic profile

$$\begin{array}{c} {\bar{u}}_{z}=\frac{u_{*}}{\kappa }\ln(\frac{z}{z_{0}}), \end{array}$$ (10)

where ${\bar{u}}_{z}$ is the average wind velocity (*ms*^−1^) at height *z* (m) above the surface, u_*_ is the shear velocity (*ms*^−1^), *κ* is the Von Kármán constant (0.4) and *z*_0_ is the roughness length (m). The roughness length was determined based on a terrain type classification [44] and lies between 0.0002 m (open sea, regardless of wave height) and 0.005 m (smooth surface, e.g beach). The roughness length for the IJmuiden station must be in between these values but tends slightly towards the “open sea” classification since the fetch of the onshore winds is mainly over the open sea. Using a typical roughness length of 0.001 m [2, 17], this results in

$$\begin{array}{c} {\bar{u}}_{0.9_{regional}}=\frac{\ln(\frac{0.9}{0.001})}{\ln(\frac{10}{0.001})}\cdot {\bar{u}}_{10_{regional}}≃0.74\cdot {\bar{u}}_{10_{regional}}. \end{array}$$ (11)

Regression analysis is used to describe the relation and ratio between ${\bar{u}}_{reg}$ and ${\bar{u}}_{local}$ (both at 0.9 m). To determine these relations we used all 10-minute averaged data regardless of the locations of the ultrasonic anemometers on the beach and subdivided the data into two classes; one containing all data from the entire beach and one containing only the data of the intertidal beach, as the effect of the dune topography on the regional airflow is presumably largest close to the dune front.

**Fig 5:**
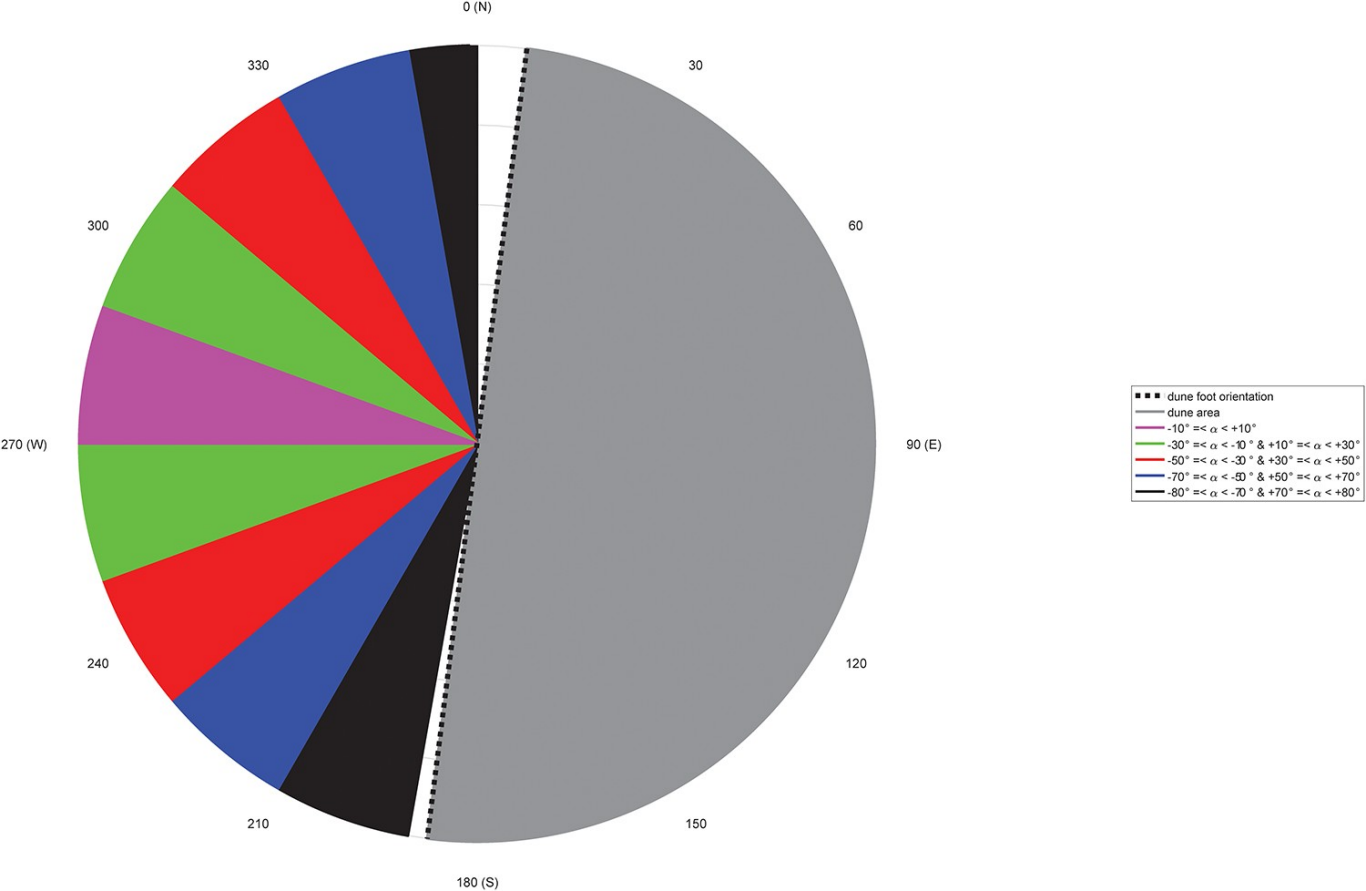
Classification of the data based on the wind direction measured in IJmuiden.

We focused on onshore approaching winds for analysis because offshore-directed winds at this site do not result in notable aeolian activity at Egmond beach [41]. The local and regional wind data have been classified based on the regional wind direction in IJmuiden and are divided into bins comprising 20°, starting from the direction perpendicular to the orientation of the dune foot (Fig 5). Classes comprising blocks of 20° enables a sufficient amount of datapoints in each class. The wind directions were reoriented relative to this perpendicular cross-shore line (277.2° from N), where 0° are the perpendicularly approaching winds and +90° and -90° are the alongshore winds from the north and the south, respectively. Here, exclusively alongshore winds (+80° to +90° and -80° to -90°) are not taken into account because of edge effects of the foredune. Such winds are normally steered directly alongshore by the foredune topography [27, 45].

To define the downwind distance of a ultrasonic anemometer relative to the dune foot, the wind direction measured in IJmuiden was used to project the distance of the ultrasonic anemometers on the dune foot orientation line (crossing the ultrasonic anemometer at the dune foot) with the Pythagorean theorem.

To give an indication of the effect of using local rather than regional wind data on the onshore potential sand transport rate, *q* (*kg*/*m*/*s*) at Egmond, we adapted the formula of [8] and [12],

$$\begin{array}{c} q=1.14\times 10^{-5}\cdot {\bar{u}}_{reg}^{3}\cdot \cos(\theta_{reg}). \end{array}$$ (12)

The empirical constant 1.14 × 10^−5^ was determined using measured D50 (∼250 *μ* *m*) and *z*_0_ (0.001 m) in the field. Based on previous research, described in the introduction, we expect that the wind speed reduction and alongshore steering is dependent on the approaching wind angle. So, combined with the results of the relation between the regional and local wind speed and direction, we adapted Eq (12) into

$$\begin{array}{c} q(\theta_{reg})=1.14\times 10^{-5}\cdot {\left(f(\theta_{reg})\cdot {\bar{u}}_{reg}\cdot +b(\theta_{reg})\right)}^{3}\cdot \cos(\theta_{reg}+\theta_{steering}), \end{array}$$ (13)

where the subscript *reg* indicates the use of the regional wind characteristics, here at 10 m height. In this equation, the ${\bar{u}}_{reg}$ is transformed into ${\bar{u}}_{local}$ with a linear regression depending on *θ*_*reg*_; and in the cos-term the *θ*_*reg*_ angle is modified to the local angle by adding the alongshore steering.

## Results

### Wind speed

**Fig 6:**
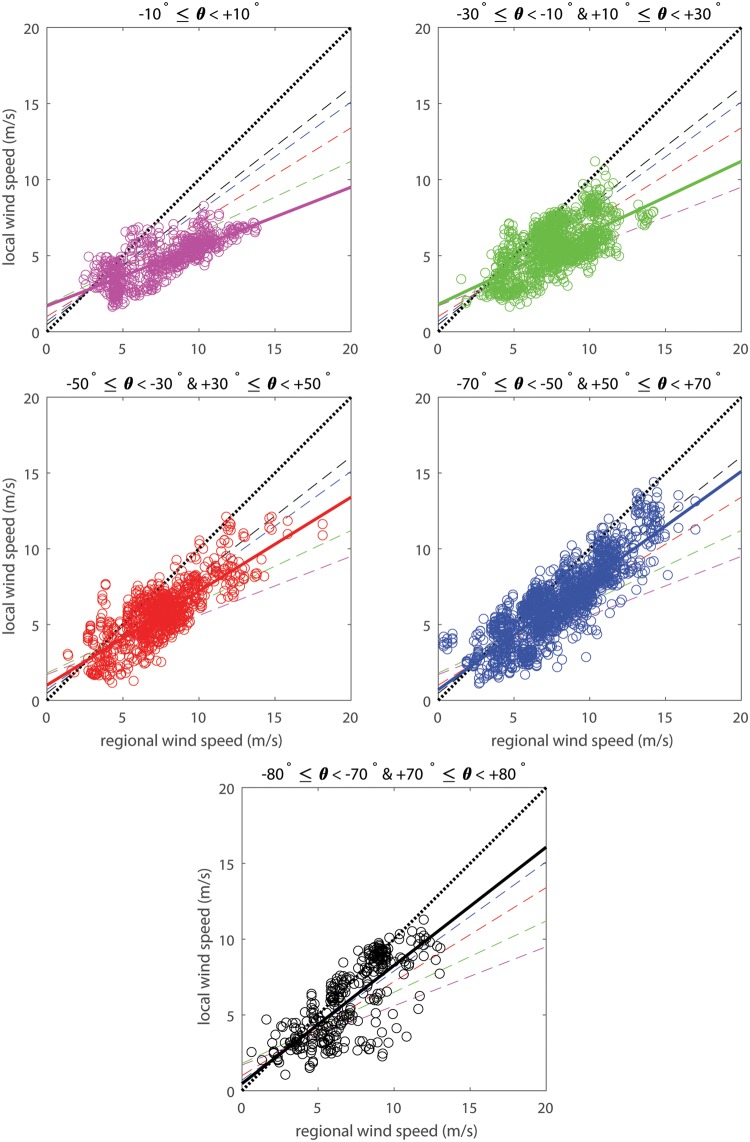
Comparison of the (regional) wind speed at IJmuiden estimated at 0.9 m height and the measured (local) wind speed at all locations at the beach near Egmond aan Zee for each 20° bin (based on the wind direction measured in IJmuiden). The colors indicate all data points in a certain 20° bin from perpendicular (manganese) towards almost alongshore (black) approaching winds relative to the dune normal. The black dotted line indicates equality of the regional and local wind speeds and the colored thick lines are the regression lines corresponding to the same-colored data points. For regression information see Table 1.

Regional wind speeds at IJmuiden transformed to 0.9 m height ranged between 0.4 and 18.2 m/s, and were compared with the local wind speeds measured at the beach in Egmond aan Zee with speed ranging from 1.0 to 14.3 m/s (Fig 6). In general, the results show that the local wind velocities are smaller than the regional wind velocities except for velocities smaller than 3.0 to 4.0 m/s. Linear regressions were used to determine the relation between the local and regional wind velocities in each of the 20° bins of the regional wind direction (Table 1). The ratio of local to regional wind velocities is smallest (0.39) during perpendicular approaching winds and increases when the wind direction is more parallel to the dune front (0.78). This ratio has a more or less steady increase from perpendicular to alongshore winds. If only the measurements on the intertidal beach, the location where aeolian transport is generally initiated, are taken into account, the ratio of local over regional wind velocities is not very different from the situation when all measurement locations are included (Table 1). The only exception is the ratio of the alongshore class (70° to 80°, both northerly and southerly winds), which approaches the value 1 and thus the local wind velocities are (almost) equal to the regional wind velocities during alongshore winds. Overall, the local over regional wind speed ratio increases with obliquity due to the change in how the wind experiences the beach-foredune morphology. When the wind becomes more alongshore directed, the slope of the morphology appears smaller and hence the local wind resembles the regional wind more.

**Table 1:** Linear regressions of the comparison of regional and local wind velocities based on the regional wind approach angle.

wind direction (°)	entire beach			intertidal beach		
	slope	y-intercept	R^2^	slope	y-intercept	R^2^
-10–+10	0.39	1.70	0.59	0.39	1.70	0.25
+/-10–+/-30	0.47	1.80	0.42	0.45	3.40	0.37
+/-30–+/-50	0.62	1.00	0.62	0.65	1.60	0.79
+/-50–+/-70	0.72	0.70	0.72	0.76	0.73	0.77
+/-70–+/-80	0.78	0.47	0.60	0.98	0.19	0.85

Linear regressions are given for data of all cross-shore locations on the entire and intertidal beach.

**Fig 7:**
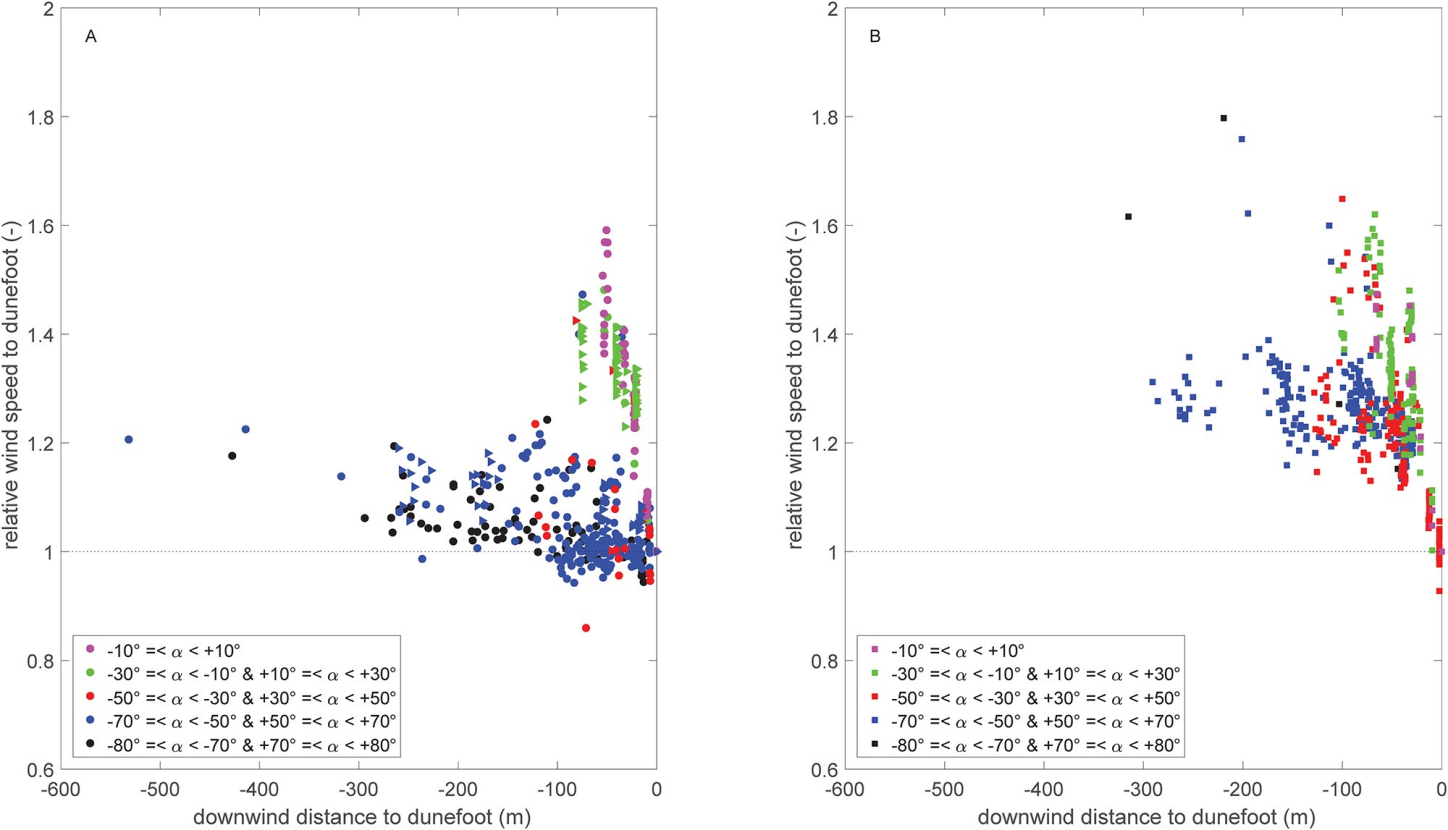
Local wind speed on the beach relative to that at the dune foot as a function of the downwind distance to the dune foot. Note that A and B show data from the two field campaigns in 2015 and 2017, respectively, and are separated due to morphological differences. The embryo dunes in front of the foredune are higher (0.8–1.0 m) in the situation of B compared to the situation of A (embryo dunes of ∼0.5 m).

Besides the general dependence on regional wind direction, a large amount of scatter is visible in the data. This scatter can partly be explained by the spatial variability in wind velocity at the beach (Fig 7). Again, a dependency on the regional wind direction can be observed. Winds approaching with angles between -30° and +30° have the largest velocities at the seaward side of the beach. This is shown in Fig 7 by a decrease in relative wind velocity in the downwind direction towards the dunefoot. During these conditions wind velocities are about 50% larger at the seaward side of the beach compared to the dune foot. During the less oblique and more parallel approaching winds (50° to 80°, both northerly and southerly winds), measured without developed embryo dunes (Fig 7a), the relative wind velocities are smaller. Here, the wind velocities at the beach are 20% larger compared to the wind velocities at the dune foot. There is thus less spatial variability at the beach compared to onshore winds. In autumn 2017, the case with embryo dunes (Fig 7b), the relative wind velocities for these wind angles are relatively higher (about 1.3), and thus wind velocites are about 30% larger at the seaward side compared to the dune foot. In nearly all cases velocities are smaller near the dune foot compared to the other locations at the beach.

### Wind direction

**Fig 8:**
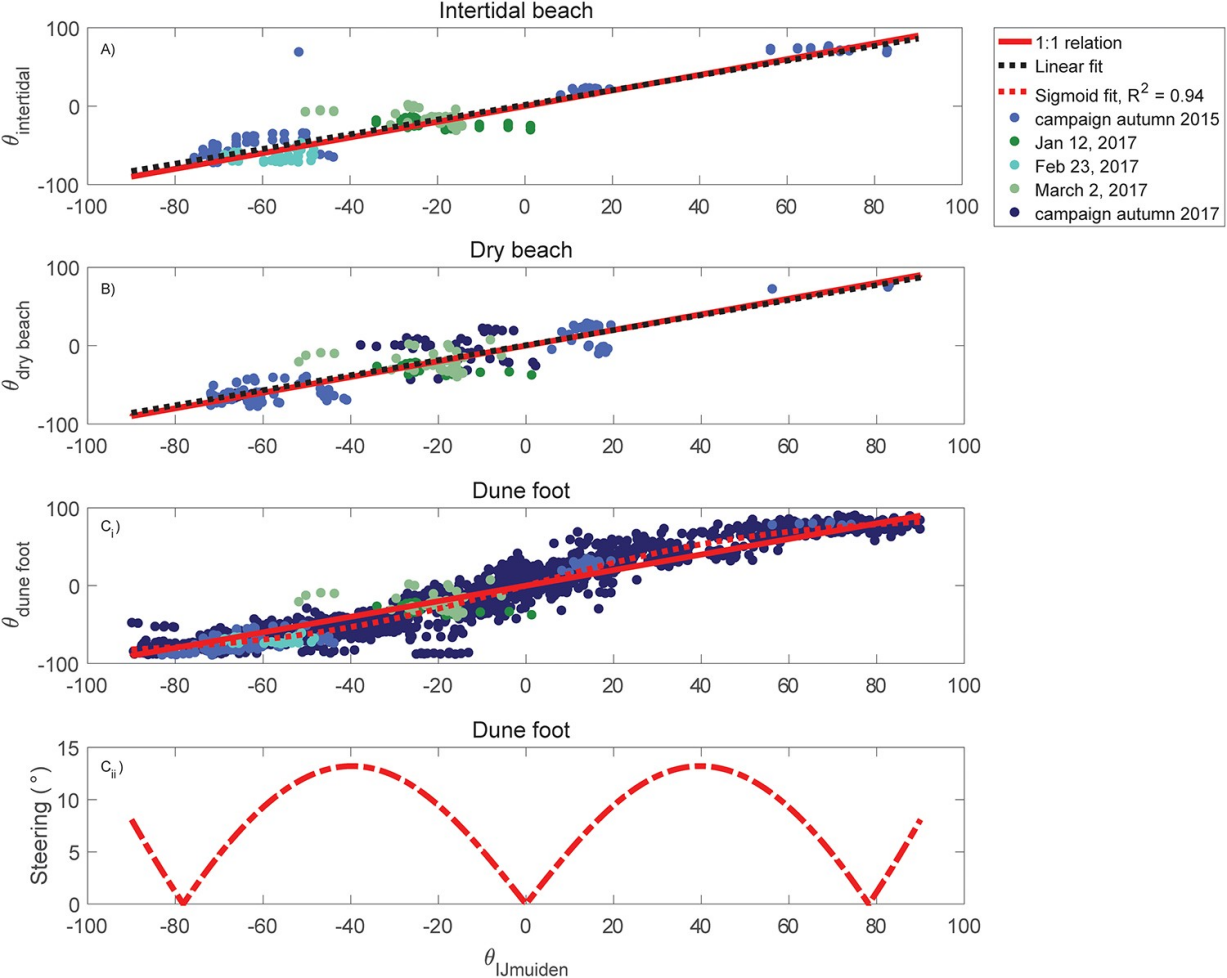
Wind steering over the beach during onshore winds. Wind direction measured at the weather station in IJmuiden is compared with the wind direction at the intertidal beach (A), dry beach (B) and dune foot (C_i_) in Egmond aan Zee. Directions are relative to the dune-normal, with negative values for southerly winds and the positive values indicate northerly approaching winds. Panel C_ii_ shows the absolute steering at the dune foot.

Fig 8A shows the relation between the regional wind direction and the wind direction at the intertidal beach when 10-minute averaged wind velocities are larger than 4 m/s. The linear fit is almost equal to the 1:1 relation, which indicates the absence of steering at the intertidal beach. The measurements at the dry beach (Fig 8B), with exclusion of the dune foot, shows a similar behaviour. At the dune foot (Fig 8C_i_) there is a more systematic deviation of the local wind direction from the regional wind direction, as found in other studies [25, 40]. During both northerly (positive directions) and southerly (negative directions) approaching winds the directions at the dunefoot are more oblique compared to the directions in IJmuiden. There is thus no 1:1 relation, instead a Sigmoid fit describes the relation with a correlation coefficient squared of 0.94. This difference can be highlighted with the absolute difference between the Sigmoid fit and the 1:1 relation, resulting in the net steering of the wind at the dune foot (Fig 8C_ii_). The absolute difference shows that during exactly perpendicular winds (0° at IJmuiden) and more or less dune-parallel (-78° and 78° at IJmuiden) approaching winds, wind steering is absent as demonstrated by [40]. The maximum steering, which is 13.2°, is observed during both 40° northerly and southerly approaching winds. During northerly winds the wind is deflected towards the south and vice versa. The increase in steering during the more alongshore approaching winds (smaller than -78° and larger than 78°) might be assigned to the boundary effects of the foredune or the presence of embryo dunes, which may cause rebound of the wind. Overall, steering is not a gradual phenomenon at the beach but can be observed predominantly just in front of the foredune.

### Potential onshore sand transport

Five different scenarios were used to quantify the effect of using the regional or local wind conditions on the magnitude of potential sand transport rate *q* (Table 2). For *f*(*θ*_*reg*_) we used the slope and for *b*(*θ*_*reg*_) we used the y-intercept of the linear regressions at the intertidal beach (right-handed side of Table 1). We computed the onshore component of the sand transport rate using the cosine effect, cos(*θ*_*reg*_), adapted in scenarios 4 and 5 with the steering component cos(*θ*_*reg*_ + *θ*_*steering*_). For *θ*_*steering*_ we used the 2 parabolas resulting from Fig 8C_ii_:

$$\begin{array}{c} \theta_{steering}\left(\theta_{regional}\right)=-0.0083{\left(\theta_{regional}+/-40\right)}^{2}+13.2, \end{array}$$ (14)

where the + /−-sign indicates steering from the north (− sign) or south (+ sign). Additionally, it was assumed that regional wind directions smaller than -78.2° and larger than 78.2° would not result in steering (*θ*_*steering*_ = 0) and that offshore directed winds do not contribute to the onshore component of *q*. The translation from *q* to annual deposition volume *Q* was made using a porosity of 0.4 and a sand density of 2650 *kg*/*m*^3^. All scenarios were run with the onshore 10-minute averaged wind data from the IJmuiden weather station between January 1, 2007 up to and including December 31, 2016.

**Table 2:** Five scenarios to compute the effect of the use of local, instead of regional, wind conditions in aeolian sand transport models.

scenario	wind velocity	wind angle	Q (*m*^3^/*m*/*y*)
1	${\bar{u}}_{regional}$	-	135
2	${\bar{u}}_{regional}$	*θ*_*regional*_	86
3	$f\left(\theta \right)\cdot {\bar{u}}_{regional}\cdot +b$	*θ*_*regional*_	40
4	${\bar{u}}_{regional}$	*θ*_*regional*_ + *θ*_*steering*_	72
5	$f\left(\theta \right)\cdot {\bar{u}}_{regional}\cdot +b$	*θ*_*regional*_ + *θ*_*steering*_	32

**Fig 9:**
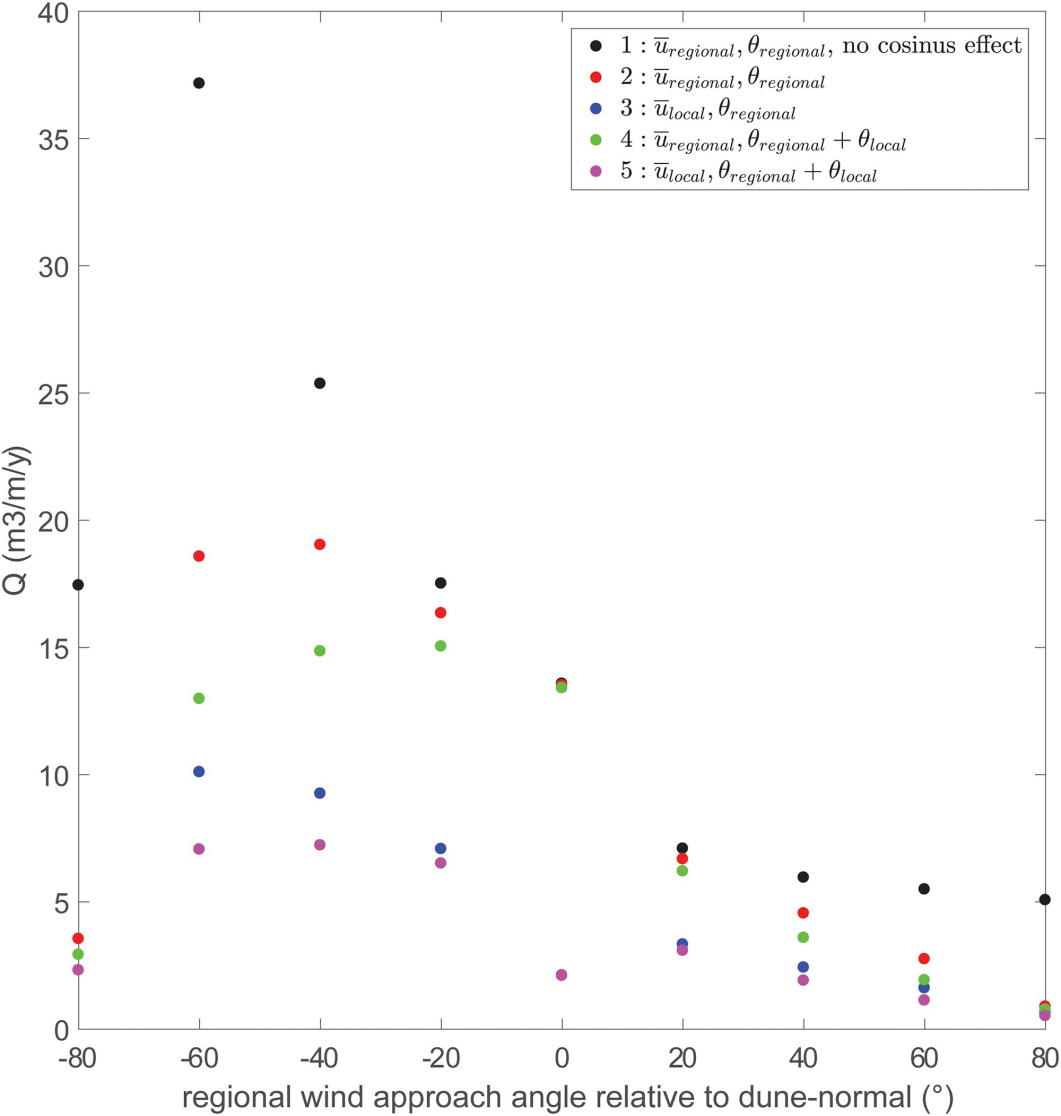
Potential transport magnitudes, based on Eq 13, in 20°-wide directional bins for 5 scenarios. The 5 scenarios are explained in Table 2.

Fig 9 shows the results of the mean transport volume per meter per year (*Q* in *m*^3^/*m*/*y*) for every approaching wind angle bin of 20°. Results show that most sand transport results from southerly approaching winds since this is the dominant wind direction (Fig 3). When the onshore component (cosine effect) is not taken into account and the regional wind is used (scenario 1, black dots), sand transport rates within a directional bin can reach up to 37 *m*^3^/*m*/*y*. However, these large transport rates are during oblique approaching winds and thus these rates (in the -60° bin) decrease considerably from 37 to about 19 *m*^3^/*m*/*y* when the cosine effect (onshore foredune supply) is taken into account (scenario 2, red dots). When ${\bar{u}}_{reg}$ is replaced by ${\bar{u}}_{local}$ (using the linear regressions in Table 1), but *θ*_*reg*_ is kept (scenario 3), the difference in sand transport magnitude is the largest especially during the perpendicular to nearly perpendicular winds. The effect of wind steering can be observed when *θ*_*steering*_ is added to the the cosinus effect (scenario 4, green dots). The decrease in transport magnitude is most pronounced with oblique approaching winds (40°—60°, both northerly and southerly), but smaller compared to the use of local instead of the regional wind speed.

The total influence of replacing regional by local wind is quantified further by taking the cumulative amount of potential sand transport of each bin, resulting in the total average annual onshore sand supply (referred to as *Q* in Table 2). As can be seen, replacement of regional by local winds causes *Q* to decrease from 86 to 32 *m*^3^/*m*/*y*, which is a drop of more than 60% and is mainly caused by the decreased wind speed. Wind steering is of secondary importance, but not negligible, and is in the order of 15–20%.

## Discussion

### Local vs. regional wind data

Although both local and regional wind velocity measurement locations have comparable surroundings, there might be differences in surface roughness because the locations are 15 kilometers apart. We assume that these differences are negligible small and have no effect on wind steering.

Local wind velocities are in general smaller than regional velocities (Fig 6) and the wind at the foredune toe is steered towards a more alongshore direction (Fig 8). However, regional wind velocities of 4 m/s and smaller are sometimes related to higher velocities on the beach (Fig 6). This turning point at low wind velocities was also observed in [25] for other locations along the Dutch coast. It might be caused by the relatively high gustiness of low wind velocities, causing a large range of velocities higher and lower than the regional wind velocities. Consequences for aeolian sand transport are not expected since the critical wind velocity for aeolian sand entrainment lies around 8 m/s at 10 m (∼ 5.9 m/s estimated at 0.9 m) based on the occurence of aeolian bed forms on Egmond beach [41]. The largest topographic steering from the dune foot to the dune crest is observed during oblique winds in a model study [40] and conceptualized in [27] based on collective empirical results which are summarized in [39]. Our work is in line with the idea, described in [27], that flow separation occurs just in front of the foredune and part of the wind is deflected into a more alongshore direction at the dune foot. According to previous studies [25, 27, 40], the wind direction returns in a more dune-normal direction further upwind at the dune front. Here we showed that steering mainly and suddenly occurs at the dune foot, whereas elsewhere on the beach steering is absent. The degree of steering, however, depends on the measuring height [40], so it is possible that closer to the bed alongshore steering near the foredune toe is stronger than observed here. Previous research [25, 26, 29] has shown that the foredune height and steepness affects the degree of influence on the airflow around a coastal foredune. It seems reasonable to expect that local wind velocities become lower and steering is larger with higher and/or steeper foredunes [46] because the build up of pressure at the dune foot would then be more significant.

Fig 7 illustrates that the reduction in wind speed toward the dune foot is rather gradual. In more detail, we do not see any clear acceleration or deceleration related to the presence of the intertidal bar and trough, respectively, as was observed by [34]. This was observed, however, on a macro tidal beach with a multiple bar-trough morphology (approx. 1 m height difference) and smaller foredunes (up to 10 m) compared to the foredunes in our study. Most likely, at the beach of Egmond aan Zee the high and steep foredune has a predominant effect on the windflow compared to the relatively small and gentle intertidal bar.

### Consequences of local wind behaviour on the magnitude of the onshore aeolian sand transport rate

Since regional weather station data are often used in sand transport models to predict dune growth, the mismatch between regional and local wind velocities and direction may result in an overestimation of the aeolian sand transport magnitude, as previously demonstrated by [11]. This mismatch is dependent on the approaching wind direction measured at the regional weather station. Despite the mainly onshore directed component of the sand transport vector during perpendicular approaching winds, a decrease in magnitude, based on the observed decrease of wind velocity, is expected. Aeolian transport due to oblique approaching winds decreases the magnitude of the onshore sand transport component since steering increases the approaching wind direction, due to deflection at the dune foot. And last, alongshore winds are the least affected by the presence of a foredune and the local winds are most similar to the regional winds, however, the onshore component of transport is in this case small due to the high obliquity.

Based on 14 surveys in 2.5 years using mobile laser scanning observations of sand deposition at the foredune indicate that the onshore annual supply at our field site is about 15 *m*^3^/*m*/*y* with an accuracy of 0.25 *m*^3^/*m* [16]. Our prediction of 32 *m*^3^/*m*/*y* indicates that the majority of the mismatch between potential and actual sand transport is due to the use of regional rather than local wind data. Our value is still a potential value and still over predicts the measured sediment supply of 15 *m*^3^/*m*/*y*. Further improvement can be expected by accounting for supply limiting factors such as moisture and weather conditions [24] or the variable beach width described by the fetch effect [20–22]. Another step in future work, together with exploring the effect of e.g. moisture and rainfall, could be to use a spatially varying, rather than a constant, wind speed in the computation of aeolian sand transport rates. Preliminary work on this [46] already shows comparable results with our measurements.

We realize that the regional to local correction factors used here are not applicable to sites with a less high and/or steep foredune. Nevertheless, our research includes an extreme morphological condition which clearly points out the effect of a large obstacle on the upwind wind conditions at the beach. The dataset, resulting from a large variety in wind directions and speeds, shows comparable results with the theoretical concept of [27] and preliminary results of Computational Fluid Dynamics (CFD) of this study area [46]. However, the regional to local correction for steering and wind speed is expected to be less profound on beaches with small and less steep foredunes. Potentially, for such other morphological conditions, correction factors for wind speed and direction can be computed with CFD, see [33]. A series of CFD runs could be processed into a lookup table with correction factors, allowing to transform time series of regional wind characteristics into local ones. As advection aeolian transport models (e.g. [47]) already contain a spatial dimension, implementation would be relatively straight forward. In critical fetch type models this could be implemented by taking a single value for the wind direction and wind speed, like we described above. Alternatively, the evaluation of the critical fetch model could be discretized along a horizontal streamline and thereby re-evaluating the critical fetch length at each spatial step based on the local wind speed and direction.

## Conclusions

Local wind conditions at the beach (speed and direction) fronting a ∼22 m high, 1:2.5 sloping foredune deviate from the regional conditions measured at a weather station ∼15 km south of our field experiments. Dependent on the regional wind direction (*θ*_*reg*_) local wind speed (${\bar{u}}_{local}$) is smaller compared to the regional wind speed (${\bar{u}}_{reg}$) and the wind direction turns into a more alongshore direction. The translation from regional to local wind conditions can be broadly subdivided into 3 categories; perpendicular onshore, oblique onshore and alongshore approaching winds. Local perpendicular onshore wind speeds are generally 39% of the magnitude of the regional wind speeds and wind steering is absent. The magnitudes of oblique onshore local wind speeds are in the order of 60-65% of the regional wind speeds and show the largest steering at the dune toe, which is about 13° more alongshore. We observed the least difference between the local and regional wind speed and direction during almost alongshore approaching winds. In this case steering is almost 0° and the magnitude of the local wind speed is between 78 and 98% of the regional wind speed.

Additional to the deviation of the local from the regional wind conditions, a spatial variability across the beach is observed in both wind speed and direction. The degree and nature of the wind deformation in this case also depends on the regional wind angle. The spatial variability of perpendicular approaching wind speeds is considerably high, wind speeds at the waterline can be 50% larger than at the dune foot. The spatial variability decreases with increasing obliquity of the approaching wind angle. During alongshore winds, wind speed is only about 10% larger at the waterline. Steering of the wind only occurs at the dune foot and not further upwind.

These differences in wind speed and direction have a significant effect on the potential annual onshore sand transport rate at our study site. When we consider the ratio in wind speed (between ${\bar{u}}_{local}$ and ${\bar{u}}_{reg}$) and direction (between *θ*_*reg*_ + *θ*_*steering*_ and *θ*_*reg*_) in the sand transport prediction, the potential sand transport rate drops from 86 to 32 *m*^3^/*m*/*y*, which is more than 60%. Since the actual annual sediment transport rate at Egmond beach is measured to be around 15 *m*^3^/*m*/*y*, the larger part of the mismatch between potential and actual sand transport rate can be explained from the use of regional in stead of local wind conditions.
